# PURPOSE PROMOTES MENTAL HEALTH FOR RETIREES, BUT THEY NEED TO HELP FINDING IT

**DOI:** 10.1093/geroni/igad104.3543

**Published:** 2023-12-21

**Authors:** Rachel Best, Anthony Ogliore, Payton Rule, Patrick Hill

**Affiliations:** Yeshiva University, Bronx, New York, United States; Saint Louis University, St. Louis, Missouri, United States; Washington University in St. Louis, St. Louis, Missouri, United States; Washington University in St. Louis, St. Louis, Missouri, United States

## Abstract

Previous research shows that sense of purpose is significantly related to mental health outcomes, including depression and anxiety (Crego et al., 2021; Dixon, 2007). Retirees are at higher risk for having a lower sense of purpose (Hill & Weston, 2019; Pinquart, 2002). Accordingly, helping retirees increase their sense of purpose may be a good way to also improve their mental health. First, research is needed to understand the relationship between sense of purpose and anxiety and depression in retirees. The current study uses cross-sectional data from 1975 adults (516 retirees and 1459 non-retirees; mean age = 49.23, SD = 18.29 ). Pearson r correlations were run between sense of purpose (Life Engagement Test), depression (PHQ-9) and anxiety (GAD-7) in retirees and non-retirees. T-tests were run between retirement status, sense of purpose, depression, and anxiety. As expected, sense of purpose was associated with lower depression (retirees r = -.33, p <.001 ; non-retirees r = -.28, p <.001) and anxiety (retirees r = -.32, p <.001 ; non-retirees r = -.29, p <.001) for both retirees and non-retirees. Results also showed that retired older adults had lower sense of purpose than older adults who are still working (t(1090) = -4.8778, p < .001). These data suggest that targeting sense of purpose in retirees is a worthwhile goal that may also indirectly improve mental health outcomes.

